# “Bones in the Medulla Oblongata?”—A Case Report of Intracranial Teratoma and Review of the Literature

**DOI:** 10.3389/fped.2021.628265

**Published:** 2021-05-05

**Authors:** Xu Zhang, Hongxiang Wang, Fan Hong, Tao Xu, Juxiang Chen

**Affiliations:** ^1^Department of Neurosurgery, Changzheng Hospital, Navy Military Medical University, Shanghai, China; ^2^Department of Neurosurgery, Changhai Hospital, Navy Military Medical University, Shanghai, China

**Keywords:** medulla oblongata, teratoma, craniotomy, neurosurgery, pediatric

## Abstract

Teratoma in the medulla oblongata is extremely infrequent and has been rarely reported. Severe and sustained brain stem compression resulting from these granitic tumors may cause potentially fatal impairment. Here, we reported a novel case of teratoma that occurred in the medulla oblongata. This 15 year-old boy suffered from a progressive gait disturbance and weakness of limbs for nearly 13 years. Magnetic Resonance Imaging (MRI) revealed an unusual mixed mass in the medulla oblongata and C1-2 spine, which was confirmed as mature teratoma by histopathological examination. Then, surgical resection was performed, followed by postoperative continuous rehabilitation. After a period of rehabilitation, this patient is currently able to mobilize with sticks. No signs of local recurrence occurred. Conclusively, surgical removal is still the preferred treatment for teratoma.

## Introduction

Intracranial teratomas, one of Central Nervous System (CNS) non-germinomatous germ cell tumors (NGGCTs), according to the 2016 CNS WHO classifications, account for 0.5% of all intracranial tumors and 2–4% of intracranial tumors in children (1). CNS teratomas commonly originate near the midline area of the brain, especially the pineal gland and suprasellar region, as solitary or multiple lesions (2). Teratomas in the brainstem are rare and may present diagnostic difficulties. Till now, only 4 cases of teratoma in medulla oblongata have been reported: one in adult and 3 pediatric patients (3–5). Surgery resection is essential but challenging. Overall, the outcome of teratoma in medulla oblongata remains poor. Prognosis varies depending on the excision of the tumor, which acts as a space-occupying lesion. Overall, the clinical outcome of teratoma in the medulla oblongata proved to be undesirable.

## Case Report

**Figure 1:**
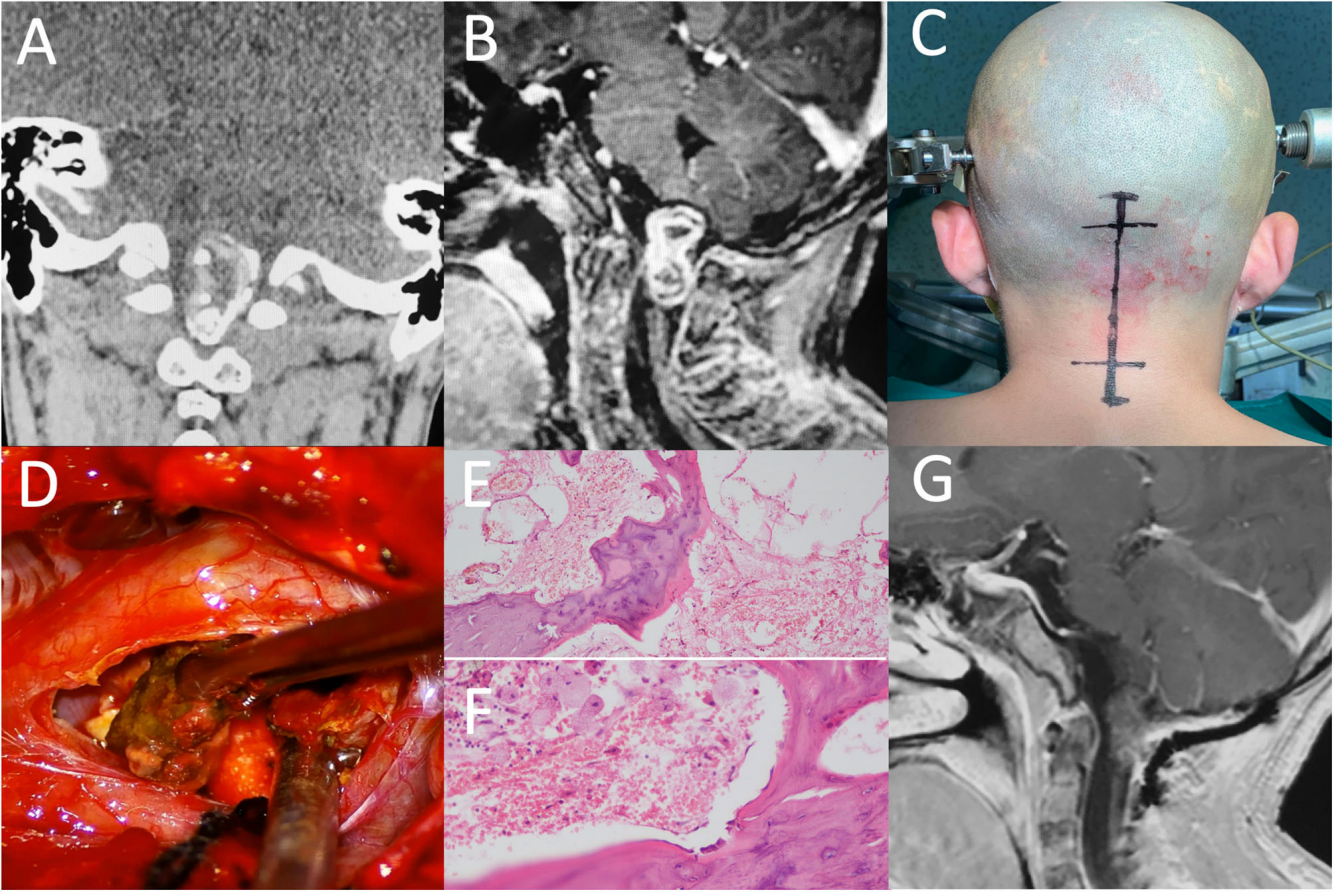
**(A)** CT of the brain demonstrates the mass filled with calcified components. **(B)** After injection of Gd, a heterogeneous enhanced lesion, extending to the medulla oblongata and C1 spine, was displayed on T1-weighted sagittal image. **(C)** A neurosurgery was performed via a midline sub-occipital approach. **(D)** Osteoid components were removed during operation. **(E,F)** Photomicrographs showing the bone structure and lipid dropletof the lesion. **(H,E)** original magnification × 100 and × 200. **(G)** Postoperative enhanced MRI showed no residual or recurrence at three months of fellow up.

A 15 year-old boy was admitted to our hospital with a 13 year history of progressive gait disturbance and weakness of limbs. Magnetic resonance imaging (MRI) performed in 2008 showed a mass lesion with mixed signal compressing the medulla oblongata (the primary image was lost because of floods that year). However, his parents refused surgery due to high risks of neurological impairment and chose the palliative treatment of achilles tendon lengthening instead. Symptoms were slightly alleviated in the following years. One year ago, the patient received a Computed Tomography (CT) scan revealing a predominantly calcified lesion in the same area of the medulla oblongata. Subsequently, MRI examination (Figure 1) indicated a markedly large mass from the medulla oblongata to C1 spine with a size of 3.1 cm. A high signal intensity rims around a low signal intensity core on T1-weighted and a low signal intensity rims around a high signal intensity core on T2-weighted images. A heterogeneous enhanced lesion was displayed on contrast-enhanced T1-weighted images. Digital subtraction angiography (DSA) excluded the possibility of vascular malformation. No other lesions were seen in CNS. Neurological examination revealed bilateral 4/5 strength of lower limbs and increased muscle tension with normal deep tendon reflexes in both upper and lower limbers. Babinski sign was positive bilaterally, but deep and superficial sensations were normal. Preoperative biomarkers in serum including Alpha-fetoprotein (AFP, 1.02 ug/L) and beta subunit human chorionic gonadotropin (β-HCG, 0.1 U/L) appeared in low levels.

The patient underwent a craniotomy via a midline sub-occipital approach in prone position. A vertical skin incision was made from the external occipital protuberance to the C2 spinous process. The foramen magnum was opened and then C1-2 laminectomy was performed. Intraoperatively, a slightly swollen medulla oblongata was exposed. An ossified crust surrounded by soft material and partial calcified portion were visible. We carefully isolated the tumor border from the brain stem and performed a complete removal of the intramedullary lesion. The patient tolerated the procedure well without any neurological deterioration.

Pathological examination (Figure 1) found well-differentiated bone tissue, some squamous epithelium and fatty tissue within the mass. A final diagnosis of mature teratoma based on neuro-imaging findings and maturely differentiated components was carefully made by three different experienced pathologists.

Postoperatively, the strength of the right limbs recovered to 4+/5 with normal muscle tone, while the left remained as it was. Then, the boy received hyperbaric oxygen therapy and exercise-based rehabilitation, involving skin care, muscle massage, and passive mobility training of left limbs. During the follow-up period of 1 year, the strength of the left limbs had improved progressively. Currently, he is able to walk independently without any evidence of tumor recurrence.

## Discussion

Intracranial teratomas are comprised of mature cells that originate from three embryonic layers: ectoderm, mesoderm, and endoderm. During the 3rd to 4th week of embryonic development, neural tube closes and these tissue implantation leads to cyst formation. Some misplaced cells of the embryonic disc, may be involved in this movement and migrate with the moving cells of the mesoderm, especially the lateral mesoderm, to the future cranial area (6). Our case was pathologically proved to be a mature teratoma because of containing well-differentiated components from mesoderm (bone and fatty tissue) and ectoderm (squamous epithelia). Occasionally, they could develop from a single germ layer if they showed histologically divergent differentiation. Ijiri et al. described a case of spinal mature teratoma, whose components were from only the mesoderm layer (7).

The World Health Organization has classified teratoma into the following groups (1): mature teratoma, immature teratoma, and teratoma with malignant transformation. Some researchers have found that biomarkers like β-HCG and AFP can be used for distinguishing mature teratomas with immature or malignant components (8). These marker proteins are sometimes secreted by immature teratomas or embryonal carcinomas that mature teratomas usually present negatively. While other immunohistochemical markers like PLAP and c-kit were not particularly helpful in obtaining a differential diagnosis of histological subtypes (9). Masao et al. determined the serum titers of AFP and β-HCG in 7 patients with intracranial mature teratomas and found that none of them had elevated titers, which was consistent with us (10).

As midline lesions, these tumors usually occupy the pineal and suprasellar region (2). Rarely, they have been described in the fourth ventricle (11, 12). Other non-midline sites that have been reported are lateral ventricles, basal ganglia, cerebellopontine angle, and cavernous sinus (13–15). Given variations in size and location of tumors, preoperative signs and symptoms are different from each other (14). Previous cases show that gait disturbance is the most common symptom (3 of 5), followed by headache (1 of 5) and paroxysmal vomiting (1 of 5), which may have been a symptom of intermittent raised intracranial pressure. MRI imaging often displays heterogeneous mass with cystic components on T1 and T2 weighted images dependent on the contents within tumors (16). CT scan can sensitively demonstrate regions of calcification in most teratomas (13, 17).

Teratoma in the medulla oblongata is extremely infrequent. According to our extensive literature review, only four cases of primary teratoma in medulla oblongata had been reported so far (Table 1) (3–5). Combined with our novel case, a total of five patients diagnosed with teratoma in the medulla oblongata were all male in Eastern Asia. Eighty percent (4 of 5) of them were children at the initiation of treatment aged in the range of 9–15 years old (mean 12 years), except a 39 year-old adult diagnosed with malignant teratoma reported by Tsuzuki et al. (5). Sixty percent of patients (3 of 5) have got the gross total resection, and the others underwent chemo-radio-therapy after subtotal resection. Based on the particularly high risk of neurosurgical removal, follow up of these five cases has shown poor prognosis. Unfortunately, 2 of them died 3 months after treatment, because of the intracranial infection or tumor regrowth.

**Table 1:** Literature review of reported cases of primary teratoma in medulla oblongata.

**Investigator**	**Age**	**Gender**	**Size (cm)**	**Operation**	**Pathology**	**Outcome**	**Symptom**
Koh et al. (3)	14 years old	Male	Unknown	Subtotal resection and chemoradiotherapy	Mixed-GCT: 95%mature teratoma and 5% germinoma	Alive after 12 mons	Left hemiparesis, gait disturbance, and multiple cranial nerve palsies for 10 days
Tsuzuki et al. (5)	39 years old	Male	Unknown	Subtotal resection and chemoradiotherapy	Malignant teratoma	Died after 3 mons	Progressive gait disturbance for 3 mons
Li et al. (4)	9 years old	Male	1.4*0.8*1.0	Total resection	Mature teratoma	Died after 1 mon	Neck pain and repeated paroxysmal vomiting for 6 mons
Li et al. (4)	10 years old	Male	3.4*3.6*3.2	Total resection	Mature teratoma	Alive at 59 mons	Intermittent headache for 1 mon
Present case	15 years old	Male	3.2*3.1*2.8	Total resection	Mature teratoma	Alive at 15 mons	Gait disturbance and weakness of limbs for 13 years

Until now, the aim of treatment always be radical resection of tumors, and postoperative adjuvant treatment for intracranial teratomas has remained controversial. Lee et al. and Bi et al. both hold the opinion that mature teratomas may be resistant to chemoradiation and could eventually grow during adjuvant therapy (18, 19). As a result, a second-look surgery would be effective for treatment of teratomas resistant to adjuvant therapy. In this case, the patient achieved gross total resection, and pathological findings indicated to be mature teratoma. We followed up closely after the operation, so far, no evidence of tumor recurrence has been found. However, the recurrence rate of immature teratomas and teratomas with malignant transformation is higher than that of mature teratomas (20). Chemoradiotherapy for immature teratomas appears to have good results, such as PE, PVB, ICE, and NGGCT regimen (18). According to some reports, combinations of high-dose radiation therapy (44–60 Gy) and chemotherapy seem to be effective for malignant teratomas (10). Therefore, for those tumors which show immature or malignant component, postoperative adjuvant treatment should be reserved.

## Limitations

Our study has some limitations. Although, there was no positive biomarkers in serum that could make sure of mature teratoma specifically, we failed to detect the relevant markers in patient's CSF, which may have missed some meaningful results. However, the gold standard still depends on the postoperative pathological findings. Furthermore, his parents had been delayed for many years to decide the operation, and the symptoms developed to a more serious situation, otherwise he may got a better outcome. Currently, this study was a retrospective study performed in a single case, and the follow-up duration was not long enough for benign tumors. As well as the extremely low incidence of this disease, there was ineluctable statistical deviation in the literature review. It is necessary to conduct related cases in the future for further summary.

## Conclusion

This study retrospected an extremely rare case of mature teratoma in the medulla oblongata. Bsed on our experience, early surgical resection with the capsule maintained intact is a preferable option before irreversible neurological dysfunction occurs. The surgeon needs to maintain the balance between the degree of tumor resection and the preservation of neurological function. Also, a long-term follow-up and further summary of related cases in the future is required to obtain a definite prognosis. Finally, further studies focusing on biological characteristics are needed, which can help the management of this tumor in the medulla oblongata.

## Data Availability Statement

The datasets presented in this study can be found in online repositories. The names of the repository/repositories and accession number (s) can be found in the article/Supplementary Material.

## Ethics Statement

Written informed consent was obtained from the individual (s), and minor (s)' legal guardian/next of kin, for the publication of any potentially identifiable images or data included in this article.

## Author Contributions

XZ, TX, and JC: conception or design of the work. XZ, HW, and FH: drafting the paper. TX and HW: revising it critically for important intellectual content. TX and JC: provide approval for publication of the content. All authors contributed to the article and approved the submitted version.

## Conflict of Interest

The authors declare that the research was conducted in the absence of any commercial or financial relationships that could be construed as a potential conflict of interest.
